# Cholangitis Due to Candidiasis of the Extra-Hepatic Biliary Tract

**DOI:** 10.1155/1998/75730

**Published:** 1998-08

**Authors:** J. D. Wig, Kartar Singh, Y. K. Chawla, K. Vaiphei

**Affiliations:** 1 Department of Surgery Postgraduate Institute of Medical Education and Research Chandigarh 160012 India; 2 Postgraduate Institute of Medical Education and Research Chandigarh 160012 India; 3 Hepatology Postgraduate Institute of Medical Education and Research Chandigarh 160012 India; 4 Postgraduate Institute of Medical Education and Research Chandigarh 160012 India

## Abstract

A case of isolated candidal fungal balls in the common bile duct causing obstructive jaundice and cholangitis is described. There were no predisposing factors. The fungal balls were removed from the common bile duct and a transduodenal sphincteroplasty was performed. *Microscopic analysis yielded colonies of candida*. Postoperative period was uneventful. At follow-up no evidence of candida infection was evident. *He is now 3 years post-surgery and is well*.


*HPB Surgery*, 1998, Vol. 11, pp. 51-54
Reprints available directly from the publisher
Photocopying permitted by license only

© 1998 OPA (Overseas Publishers Association) N.V.
Published by license under
the Harwood Academic Publishers imprint,
part of The Gordon and Breach Publishing Group.
Printed in India.

*Case Report*

### Cholangitis Due to Candidiasis of the Extra-Hepatic Biliary Tract

J. D. WIG[a,*], KARTAR SINGH[b], Y. K. CHAWLA[c] and K. VAIPHEI[d]

[a] *Department of Surgery, Postgraduate Institute of Medical Education and Research, Chandigarh - 160012, India;*
[b] *Gastroenterology, Postgraduate Institute of Medical Education and Research, Chandigarh - 160012, India;*
[c] *Hepatology Postgraduate Institute of Medical Education and Research, Chandigarh - 160012, India;*
[d] *Pathology, Postgraduate Institute of Medical Education and Research, Chandigarh - 160012, India*

*(Received 10 May 1996; In final form 28 February 1997)*

**A case of isolated candidal fungal balls in the common bile duct causing obstructive jaundice and cholangitis is described. There were no predisposing factors. The fungal balls were removed from the common bile duct and a transduodenal sphincteroplasty was performed. *Microscopic analysis yielded colonies of candida.* Postoperative period was uneventful. At follow-up no evidence of candida infection was evident. *He is now 3 years post-surgery and is well.***

*Keywords:* Biliary candidiasis, fungal balls, cholangitis, nasobiliary drainage

### INTRODUCTION

Candidiasis of the extrahepatic biliary tract is rare [1,2,3] and can involve the biliary tract either as a component of a systemic infection, or as an isolated affliction. We describe a patient with candidial jaundice in whom there were no apparent predisposing factors and candida resulted in the formation of obstructive fungal balls without any other signs of organ involvement.

### CASE REPORT

A 73 years male presented with colicky right upper abdominal pain of two months duration, progressively deepening jaundice of one month duration, associated with intense pruritis and fever of 5 days duration. On examination, he was deeply jaundiced. There were no signs of hepatic encephalopathy. There was firm liver edge 4 cm below the costal margin with smooth surface. The possibilities were either a stone in common bile duct or a periampullary tumour.

Investigation showed a haemoglobin of 10.8 g/dl with a normal leucocyte count. Total serum bilirubin 219.3 μ mol/L with conjugated 171.4

*Corresponding author.

μ mol/L, alkaline phosphatase 197.8 IU/L, SGOT 62 IU, SGPT 60 IU, total proteins 70 g/L, albumin 43 g/L, globulin 27 g/L, prothrombin index 82 percent: blood urea 7.53 mmol/L and serum creatinine 132.7 μ mol/L. An ultrasound examination revealed extra-hepatic biliary obstruction and the exact cause of which could not be identified. Liver and pancreas were normal. On endoscopic retrograde cholangiopancreatography (ERCP), Papilla was hyperaemic and oedematous and the cholangiogram demonstrated radiolucent filling defects in distal part of the common bile duct. The gallbladder was not visualised. *Cholangiographic appearance was not suggestive of a common bile duct cyst.* A nasobiliary drainage was instituted at the time of ERCP (Fig. 1). Bile obtained from nasobiliary collections grew no organisms.

The patient was explored twelve days after institution of the nasobiliary drainage. The gallbladder was absent. *There were no other anatomical abnormalities of the biliary tract.* Dense adhesions were present in the region of common bile duct, which could not be identified in the supraduodenal part. The duodenum was opened. The nasobiliary catheter acted as a probe and a papillotomy was performed. The papilla was oedematous and friable. Membranous material (Fig. 2) was evacuated from the common bile duct. No stones were detected. A transduodenal sphincteroplasty was performed. The common bile duct was then opened in the supraduodenal part and was found to be thick walled and oedematous. *There was no cystic dilatation of the common bile duct.*

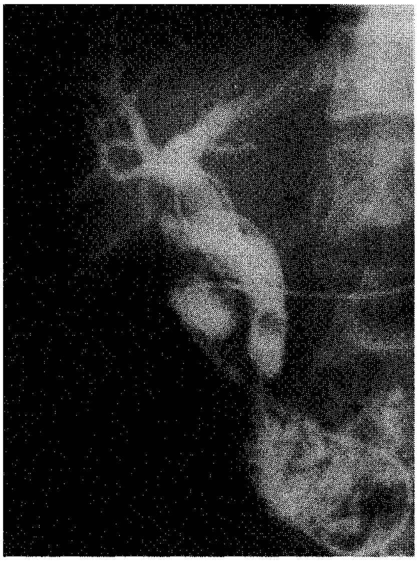

FIGURE 1 Endoscopic retrograde cholangiogram shows filling defects in distal part of common bile duct.

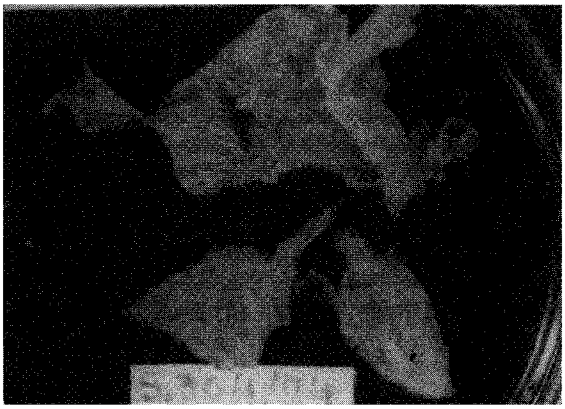

FIGURE 2 Membranous material removed from the common bile duct.

Patency of the right and left hepatic ducts was ensured after irrigating them with normal saline. The common bile duct was closed over a T-tube for drainage. A completion cholangiogram was found to be normal, thereby ensuring the patency of hepatic ducts and common bile duct. Bile cultures taken at operation revealed growth of citrobacter species and streptococcus faecalis.

Histopathological examination of the membranous material yielded colonies of fungal hyphae and bacterial colonies in a background of amorphous fibrinous material. The morphology of fungal hyphae was consistent with that of candida and the bacterial colonies were found to be gram positive organisms on special stain.

The postoperative period was uneventful and patient had a complete recovery. Repeated examinations of bile failed to reveal any fungi. Fungal serology was also negative. No antimycotic therapy was given in the postoperative period. There was no evidence of systemic candida infection.

The final diagnosis was isolated candidiasis of common bile duct causing extrahepatic cholestasis.

At follow up the liver functions were normal, total serum bilirubin 17.1 μ mol/L, SGOT 19 IU, SGPT 7 IU, alkaline phosphatase 55.2 IU/L. A T-tube cholangiogram was normal. *He is now 3 years post surgery and is asymptomatic.*

### DISCUSSION

Fungal infections of the hepatobiliary system are uncommon and the various fungi reported to infect the biliary tree include candida albicans [1, 4, 5], blastomyces dermatitidis [6], and cryptococcus neoformens [7]. The pathogenesis is not certain-the reported postulations are direct invasion of the common bile duct from the duodenum [4, 5] or systemic candida sepsis could establish microabscess in liver parenchyma following movement of candida organisms into the bilary tree [4]. In our patient, a haematogenous route seems unlikely as there were no signs of systemic candidiasis. In a review of nine patients with candidiasis of the extra-hepatic biliary tract, systemic candidiasis was associated in only one patient [2].

Several predisposing factors for candida infection are immunocompromised patient, malignant haematologic disease, broad-spectrum antibiotic administration, corticosteroid therapy, diabetes, previous surgery or trauma [1, 2, 8, 9]. None of these factors were present in our patient. Carstensen *et al.* [5] and Marcucci *et al.* [10] have also reported common bile duct obstruction secondary to infection with candida and in their patients also there were no apparent predisposing factors.

The spectrum of biliary involvement by candida includes fungal balls in the extrahepatic biliary tree [1, 2, 4, 5] and inflammatory changes of the biliary tree [2, 11]. Our patient presented with cholangitis. The main findings were those of a cholestatic liver profile, ductal dilation with filling defects on ERCP suggestive of common bile duct stones. In a review of nine patients with candidiasis of the extra-hepatic biliary tract [2], jaundice was the clinical manifestation in three patients, fungal balls in the common bile duct were present in three patients, gangrenous cholecystitis in two, two patients had acute cholecystitis and purulent bile was present in two patients. In our patient there was congenital absence of the gallbladder and dense adhesions were present in the area of common bile duct and it could not be identified. We had to open the duodenum first, the nasobiliary catheter acted as a probe and helped in performing papillotomy and subsequently sphincteroplasty facilitated removing the fungal balls from the common bile duct. The common bile duct was then flushed with normal saline to achieve clearance, though intraoperative choledochoscopy is the most effective method for biliary tract exploration and gives the surgeon more security in complete clearance of the common bile duct [12, 13]. The common bile duct was extremely thick walled and oedematous. A completion cholangiogram had shown that the common bile duct was clear and there was free flow of contrast into the duodenum.

The treatment depends on the operative findings. We performed a transduodenal sphincteroplasty and T-tube drainage of the common bile duct after removing the fungal balls. We did not administer antifungal therapy as we thought that in the absence of any systemic candidiasis, drainage may be sufficient. Marcucci *et al.* [10] and Gupta *et al.* [1] also suggest that timely relief of obstruction to bile flow and improvement in nutritional status helps the patient to overcome the fungal proliferation. Some workers have given chemotherapy following removal of fungal balls. Treatment is highly effective with most reported patients having completely recovered. In a review of nine patients by Irani and Truong [2], two patients died of unrelated causes. Our patient had an uneventful post-operative period, and fungal serology and repeated bile cultures were negative for fungus.

### *References*

[1] Gupta, N. M., Chaudhary, A. and Talwar, P. (1985). Candidial obstruction of the common bile duct. *Br. J. Surg.*, **72**, 13.
[2] Irani, M. and Truong, L. D. (1986). Candidiasis of the extrahepatic biliary tract. *Arch. Pathol. Lab. Med.*, **110**, 1087–90.
[3] Schreiber, M., Black, L., Noah, Z. *et al.* (1982). Gallbladder candidiasis in a leukemic child, *AJDC*, **136**, 462–463.
[4] Magnussen, C. R., Olson, J. P., Ona, F. V. and Graziani, A. J. (1979). Candida fungus balls in the common bile duct. *Arch Intern. Med.*, **139**, 821–822.
[5] Carstensen, H., Nilsson, K. O., Nettelblad, S.-C., Cederlund, C.-G. and Hil'dell, J. (1986). Common bile duct obstruction due to an intra-luminal mass of candidiasis in a previously healthy child. *Pediatrics*, **77**, 858–861.
[6] Ryan, M. E., Kirchner, J. P., Sell, T. and Swanson, M. (1989). Cholangitis due to Blastomyces dermatitidis. *Gastroenterology*, **97**, 1346–9.
[7] Bucuvalas, J. C., Bove, K. E., Kaufman, R. A. *et al.* (1985). Cholangitis associated with cryptococcus neoformans. *Gastroenterology*, **88**, 1055–9.
[8] Smith, C. B. (1985). Candidiasis: Pathogenesis, host resistance and predisposing factors. In: G. P. Bodey , V. Feinstein (Eds.); Candidiasis. New York, press, pp. 53–70.
[9] Young, R. C., Bennet, J. E., Geelhoed, G. W. *et al.* (1974). Fungemia with compromised host resistance. A study of 70 cases. *Ann Intern Med.*, **80**, 605–612.
[10] Marcucci, R. A., Whitely, H. and Armstrong, D. (1978). Common bile duct obstruction secondary to infection with candida. *J. Clin. Microbiol.*, **7**, 490–492.
[11] Uflacker, R., Wholey, M. H., Amaral, N. M. and Lima, S. (1982). Parasitic and mycotic causes of biliary obstruction. *Gastrointest. Radiol.*, **7**, 173–9.
[12] Madjov, R. and Chervenkov, P. (1993). Intraoperative choledochoscopy: the most effective method for biliary tract exploration. *Br. J. Surg.*, **80**, S7.
[13] Ramirez, P., Parrilla, P., Bueno, F. S. *et al.* (1993). Long-term results of surgical sphincterotomy in the treatment of choledocholithiasis, *Surg. Gynecol. Obstet.*, **176**, 246–50.

### COMMENTARY

This report by Dr. J. D. Wig and coworkers presents a case with obstructive jaundice due to colonisation of the common bile duct with Candida. No other reason of obstruction was found but candidal fungal balls in the bile duct. The patient, however, had an agenesis of the gallbladder.

It is earlier demonstrated that biliary infection and infestation including Nematodes like Ascaris lumbricoides and Trematodes like fasciola may obstruct the bile ducts. It is also demonstrated in earlier reports that fungal infections may interfere with the drainage of bile to the duodenum. Candida albicans is not infrequently present in the biliary tract in patients with gallstones [1] or biliary stents [2]. Further, Candida infections may be a problem after liver transplantation [3]. The present study, as well as several other reports, show that Candida may cause biliary obstruction. Usually this condition is associated with a reduced host resistance but it may also appear in otherwise healthy individuals. In the present case there were no evident underlying pathology even if the congenital absence of the gallbladder may indicate that other abnormalities may be present.

### References

[1] Gupta, B. K., Kumar, R., Kaur, S. and Khurana, S. (1993). Incidence of fungal infection in extra hepatic biliary stone disease. *Indian Journal of Pathology & Microbiology*, **36**, 110–112.
[2] Yu, J. L., Andersson, R. and Ljungh, A. (1996). Protein adsorption and bacterial adhesion to biliary stent material. *J. Surg. Res.*, **62**, 69–73.
[3] Wade, J. J., Rolando, N. and Hayllar, K. (1995). Philpott-Howard, J., Casewell, M. W. and Williams, R. Bacterial and fungal infections after liver transplantation: an analysis of 284 patients. *Hepatology*, **21**, 1328–36.

Joar Svanvik
Dept. of Biomedicine and Surgery
Linköping University, Sweden

